# Presumptive steroid-responsive radiculoneuritis in dogs

**DOI:** 10.1093/jvimsj/aalag091

**Published:** 2026-05-21

**Authors:** Carmen Manuela Stan, Matthew James, Mark Lowrie

**Affiliations:** Department of Neurology and Neurosurgery Service, Pride Veterinary Referrals, IVC Evidensia, Derby DE24 8HX, United Kingdom; Department of Neurology and Neurosurgery Service, Linnaeus, Wear Referrals, Bradbury, Stockton-on-Tees TS21 2ES, United Kingdom; Department of Neurology and Neurosurgery Service, Movement Referrals, Independent Veterinary Specialists, Uttoxeter ST14 8AZ, United Kingdom

**Keywords:** dogs, German Shepherd, presumptive immune-mediated neuropathy, presumptive steroid-responsive radiculoneuritis, spinal nerve and nerve roots

## Abstract

**Background:**

Presumptive steroid-responsive radiculoneuritis (PSRRN) in dogs is a rare condition that is often difficult to distinguish from neoplasia due to overlapping clinical and imaging features. Differentiating between these etiologies is critical to determining prognosis.

**Hypothesis/Objectives:**

To describe the clinical presentation, diagnostic findings, magnetic resonance imaging (MRI) characteristics, treatment, and outcomes in dogs with PSRRN affecting spinal nerves and nerve roots.

**Animals:**

Fifteen dogs presented to 2 UK referral centers between 2019 and 2025.

**Methods:**

Retrospective review of dogs with MRI findings consistent with nerves and nerve roots inflammation involving the C6-T2 or L4-S3 spinal cord segments and a minimum clinical follow-up of 12 months.

**Results:**

Dogs had a mean duration of 8 weeks (range 4 days-24 weeks) of lameness and monoparesis, and a mean age of 50.6 months (range 12-104 months); 12/15 were male (6 neutered) and 3/15 female (2 neutered). German Shepherd dogs accounted for 4/15 cases. Magnetic resonance imaging consistently revealed contrast-enhancing, T2W, and short τ inversion recovery hyperintense lesions, frequently affecting the L7 and T1 nerves and roots. Lumbar cerebrospinal fluid analysis revealed albuminocytological dissociation in 4/5 dogs. All dogs responded to medical treatment, although 3/15 experienced relapse over a median follow-up of 25 months (range 12-50 months).

**Conclusions and clinical importance:**

Presumptive steroid-responsive radiculoneuritis should be considered in dogs with diffuse enlargement of spinal nerves and nerve roots and chronic monoparesis. Magnetic resonance imaging findings, while suggestive, are not pathognomonic and highlight the importance of considering inflammatory etiologies in the differential diagnosis of spinal nerve enlargement.

## Introduction

Diffuse enlargement of spinal nerves and nerve roots has been infrequently reported in dogs, and its clinical significance remains poorly defined. Most reports describe focal or segmental lesions, frequently associated with neoplasia,^1,2^ although presumptively immune-mediated neuritis^3–5^ has also been described and associated with spinal cord compression.^3,4^ In contrast, diffuse, noncompressive enlargement involving the entire nerve and nerve roots is not well characterized, and the underlying etiology, clinical course, and response to treatment are unclear. This knowledge gap is clinically relevant, as identification of a potentially treatable inflammatory condition may influence diagnostic and therapeutic decision-making.

We hypothesized that dogs with diffuse enlargement of spinal nerves and nerve roots in the absence of compressive features have an underlying immune-mediated origin. The objective of this study was to describe the clinical presentation, magnetic resonance imaging (MRI) findings, treatment, and outcome in a cohort of dogs with selective diffuse enlargement of spinal nerves and nerve roots localized to the C6-T2 and L4-S3 spinal cord segments, consistent with presumptive steroid-responsive radiculoneuritis (PSRRN).

## Materials and methods

A retrospective analysis of medical records was conducted using databases from 2 referral centers in the United Kingdom. Dogs presented between 2019 and 2025 were eligible for inclusion.

The inclusion criteria were dogs of any age, breed, sex, or neuter status, with clinical signs of neurological disease consistent with C6-T2 or L4-S3 spinal cord segments neuroanatomical localization, with an MRI scan showing single or multiple diffuse spinal nerve and nerve roots enlargement characterized by T2-weighted (T2W) and short τ inversion recovery (STIR) hyperintensity with or without contrast-enhancement and with a minimum follow-up period of 12 months.

The exclusion criteria included dogs with degenerative changes or other vertebral column changes on MRI or computed tomography (CT) that could have explained a secondary neuritis, as well as dogs with more focal nerve root enlargement, which is more indicative of neoplasia.

Data were retrieved from medical records, including signalment, clinical history, presenting complaint, findings from clinical and neurologic examinations and MRI, and treatment protocols and outcomes. Results of ancillary diagnostics were reviewed where available.

The onset of clinical signs was categorized as hyperacute (<24 h), acute (1-7 days), subacute (7-14 days), or subtle progressive (>15 days). Age groups were defined as young (<3 years), middle-aged (3-9 years), or old (≥10 years). Body size was classified as small (<10 kg), medium (10-25 kg), large (26-45 kg), or giant (>45 kg).

The clinical presentation was characterized based on the onset and progression of clinical signs of neurological disease, the presence of pain, lateralization, and neuroanatomical localization. All clinical and neurological examinations were performed by a board-certified neurologist or a resident under the direct supervision of a board-certified neurologist.

Computed tomography scans were performed using a 16-slice CT machine (Aquilion RXL; Toshiba Medical Systems Corporation, Tokyo, Japan). High-field MRI was conducted using either a 1.5 T Siemens MAGNETOM Sempra (Erlangen, Germany) or a Canon ELAN VANTAGE (Venlo, Netherlands) system.

Information regarding the CT scanner used at the external facility was not available.

Follow-up information was obtained from clinical records and phone conversations with the owner or referring veterinarian, including physical re-examination findings and client communications.

The data were analyzed descriptively, and no statistical analyses were performed.

## Results

Fifteen cases met the inclusion criteria. The mean age at presentation was 50.6 months (range, 12-104 months; median 40 months). Seven dogs were classified as young, and 8 dogs were classified as middle-aged. The cohort consisted of 12 males (80%) and 3 females (20%). Among males, 6 were neutered (50%), and among females, 2 were spayed (67%).

The mean body weight was 27 kg (range, 10-40 kg; median, 30 kg). Seven dogs (47%) were classified as medium-sized, and 8 dogs (53%) as large-breed.

German Shepherd dogs were the most commonly represented breed (4/15, 27%). Other breeds included one each of the following: Labrador Retriever, Labradoodle, crossbreed, Golden Retriever, Corgi-Collie mix, Flat-Coated Retriever, English Springer Spaniel, Cocker Spaniel, Pointer, Alaskan Malamute, and Staffordshire Bull Terrier.

### Clinical presentation

The mean duration of clinical signs before presentation was 8 weeks (range, 4 days-24 weeks). One dog presented with acute-onset (4 days) of clinical signs; 3 dogs with subacute-onset (range 1-2 weeks); and 11 dogs with subtle, progressive signs (range 2.5-24 weeks).

Physical examinations revealed no abnormalities in any of the dogs.

#### Dogs presented for signs of pelvic limb abnormalities (*n* = 11)

Eleven dogs were presented primarily for pelvic limb abnormalities. Five dogs had right pelvic limb involvement, 3 had left pelvic limb involvement, and 3 had bilateral pelvic limb abnormalities. Common presenting complaints in this group included progressive lameness with reluctance to jump (*n* = 5), intermittent vocalization (*n* = 5), limb stiffness (*n* = 3), and knuckling (*n* = 2). Additional complaints included difficulty standing (*n* = 2), intermittent toe-touching (*n* = 2), bunny-hopping gait (*n* = 1), intermittent non-weight-bearing lameness (*n* = 1), and nail scuffing (*n* = 1).

On neurological examination, 2 dogs exhibited a plantigrade stance in the affected pelvic limb; 1 also had nail scuffing. Gait abnormalities included monoparesis with mild lameness (*n* = 3), persistent non-weight-bearing lameness (*n* = 1), and a stiff pelvic limb gait (*n* = 2). One dog demonstrated mild proprioceptive ataxia, and another showed intermittent bunny-hopping. Muscle atrophy was present in 3 dogs, and spinal palpation elicited hyperesthesia in 8 dogs. Postural reactions and spinal reflexes were reduced in 8 dogs, consistent with lower motor neuron (LMN) dysfunction affecting the L4-S3 spinal cord segments. Increased patellar reflexes were observed in 2 cases and were consistent with a lesion affecting the L6-L7 spinal nerve roots. In 3 dogs, postural reactions and spinal reflexes were normal at presentation despite pelvic limb gait abnormalities. In these cases, neuroanatomical localization to the L4-S3 spinal cord segment was guided by findings in the affected limb, including limb stiffness, intermittent bunny-hopping, and focal hyperesthesia on spinal palpation.

#### Dogs presented for signs of thoracic limb abnormalities (*n* = 4)

Four dogs were presented primarily for thoracic limb abnormalities, all affecting the right thoracic limb. Presenting complaints included intermittent vocalization (*n* = 3), limb stiffness (*n* = 1), and knuckling (*n* = 1). One dog presented acutely, while 2 had a subacute onset, and 1 had progressive signs.

Neurological examination revealed monoparesis with mild lameness in 1 dog, severe lameness in 1 dog, and reduced postural reactions and spinal reflexes in 3 dogs. One dog exhibited clinical abnormalities consistent with Horner syndrome, including ipsilateral miosis, exophthalmos, and protrusion of the third eyelid. This dog also had right thoracic limb lameness, with reduced postural reactions, withdrawal reflexes, and muscle tone. Given the thoracic limb abnormalities, together with an otherwise normal physical and neurologic examination, Horner syndrome was thought to be most likely affecting the second-order neurons, which supported a lesion affecting the T1-T3 nerve roots. Spinal hyperesthesia was elicited in one dog, while 3 dogs showed no pain on palpation. Muscle tone was decreased in 1 dog. Neurological findings in all 4 cases were consistent with a lesion of the right C6-T2 spinal cord segment, which accounted for the observed thoracic limb LMN abnormalities and, in 1 case, more specifically the T1-T3 nerves due to Horner syndrome.

Two dogs exhibited concurrent clinical signs of gastrointestinal disease before the onset of neurologic abnormalities. One had a history of intermittent diarrhea attributed to *Campylobacter jejuni* infection diagnosed 6 months earlier, and the other had chronic intermittent diarrhea of undetermined origin. No history of trauma was reported in any dog.

### Diagnostic testing

Diagnostic investigations varied between dogs. A complete blood count and serum biochemical profile (*n* = 2), a thyroxine (T4)/thyroid-stimulating hormone panel (*n* = 1), and creatine kinase activity (*n* = 1) were all within normal limits. C-reactive protein was assessed in 5 dogs, with abnormal values in 2 (reference interval: 0.1-10 mg/L), at 14 and 97.8 mg/L, respectively.

Lumbar cerebrospinal fluid (CSF) analysis was performed in 5 dogs. The fluid was clear and colorless with normal total nucleated cell counts (≤5 cells/μL), although protein concentration was elevated in 4 of 5 cases (≥45 mg/dL) to a mean of 68 mg/dL (48-102 mg/dL).

Infectious disease testing included serologic assessment for *Toxoplasma gondii* (*n* = 2), *Neospora caninum* (*n* = 3), and *Aspergillus* spp., as well as microbial culture (*n* = 1); all results were negative.

Electromyography (EMG) was performed in 1 dog and revealed no abnormalities.

Cross-sectional CT of the vertebral column was performed in 5 dogs and revealed no abnormalities in 3. In dog 7, incidental diffuse idiopathic skeletal hyperostosis (DISH) was noted. Dog 8 had undergone CT imaging at an external facility prior to presentation. According to the referring veterinarian’s report, there was thickening of the right L7 nerve roots with foraminal expansion. However, the images and full report were not available for review, so further details are limited.

**Figure 1:**
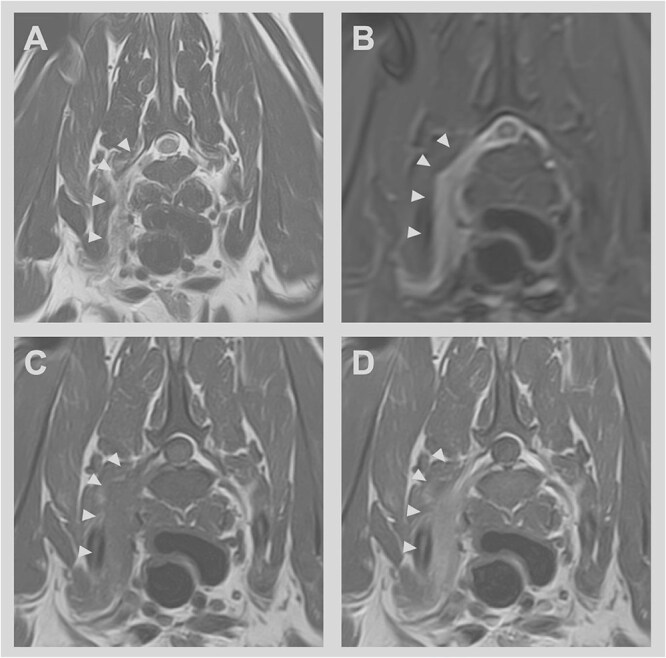
Transverse magnetic resonance images at the level of the T1 vertebral body in a 1-year-old female crossbreed dog presenting with a 1-month history of progressive pain (yelping), right forelimb nerve root signature, and right-sided Horner syndrome. The T2-weighted image (A) shows a hyperintense lesion (arrowheads) along the course of the right T1 nerve and root. The signal intensity is greater than that of the surrounding muscle but less intense than that of the adjacent fat. The lesion appears markedly hyperintense on STIR imaging (B). Transverse T1W precontrast (C) and T1W postcontrast (D) demonstrate the marked homogeneous contrast enhancement of this lesion. Abbreviations: STIR = short τ inversion recovery; T1W = T1-weighted.

Magnetic resonance imaging was performed in all 15 dogs. Protocols included T2W, STIR, and T1-weighted (T1W) sequences with and without fat saturation, as well as pre- and postcontrast T1W imaging. Affected spinal nerves and roots demonstrated diffuse enlargement, most commonly involving the right seventh lumbar (L7) (*n* = 6), right first thoracic (T1) (*n* = 3), bilateral L7 (*n* = 2), left L7 (*n* = 2), right sixth lumbar (L6) (*n* = 1), and right seventh cervical (C7) spinal nerve and roots (*n* = 1). The lesions were characterized by diffuse enlargement of the affected nerve and roots along their entire course. On T2W images, the lesions were hyperintense, with signal intensity greater than that of the surrounding muscle but less than that of the adjacent fat. Short τ inversion recovery sequences demonstrated markedly hyperintense signals within the affected nerve roots. On T1W postcontrast images, the lesions exhibited heterogeneous contrast enhancing in 1 dog, mild-to-strong homogeneous contrast enhancement in 13 dogs, and no contrast enhancement in 1 case (Figure 1).

In the 3 dogs with concurrent muscle atrophy, ipsilateral limb muscle atrophy was observed, with mild diffuse signal hyperintensity on MRI in 2 of them and not recorded in the third.

### Treatment and outcome

Initial treatment with meloxicam was initiated by the referring veterinarian in all dogs. This resulted in minimal to partial clinical improvement in thirteen cases. One dog (dog 7) showed a marked clinical response to meloxicam alone, and treatment was continued for several weeks, whereas another (dog 10) exhibited initial improvement but relapsed shortly after discontinuation and was subsequently transitioned to corticosteroid therapy. Nonsteroidal anti-inflammatory (NSAIDs) therapy was discontinued in all other dogs, and corticosteroid treatment was initiated at referral, consisting of administration of PO prednisolone, epidural methylprednisolone acetate (Depo-Medrone), or both. Five dogs with L3-S3 spinal cord segment lesions initially received 1 epidural injection (L7-S1 intervertebral space) of 1.5 mg/kg methylprednisolone acetate. Two of these dogs were treated concurrently with PO prednisolone at 0.5 mg/kg twice daily (Q12h) for 2 weeks, followed by a gradual taper over several months. One dog was treated with an initial dose of PO prednisolone at 1.6 mg/kg once daily (Q24h) for 3 weeks, followed by tapering over 4 months. Eight dogs were managed with PO prednisolone alone. Prednisolone doses ranged from 0.5 to 1 mg/kg Q12h for 1-4 weeks, with a mean tapering duration of 6 months (range 1-12 months). Adjunctive medications included gabapentin (10 mg/kg Q12h or every 8 h (Q8h) for 15-25 days) and omeprazole (1 mg/kg Q24h or Q12h for 2-4 weeks) in several cases. One dog (dog 7) received amantadine (3 mg/kg Q24h) as a rescue analgesic, in combination with clavulanate-potentiated amoxicillin (20 mg/kg Q12h for 15 days). Another (dog 6) was administered cephalexin (15 mg/kg Q12h for 4 weeks). Dogs receiving an epidural Depo-Medrone alone did not received additional analgesics.

All dogs had a favorable outcome at the end of the treatment course. Twelve dogs achieved complete clinical remission, defined as full resolution of neurological deficits, without the need for ongoing medication. Two dogs achieved clinical remission with complete resolution of neurological deficits, but they remained drug-dependent, requiring ongoing low-dose prednisolone 0.1 mg/kg Q24h in one dog or every other day (Q48h) in the other. One dog had a partial response, drug-independent (on no medication), with persistent Horner syndrome as the only residual deficit.

Relapses were recorded in 3 dogs. Dog 3 re-presented 15 months after the initial diagnosis, after 6 months of mild clinical signs. Prednisolone was restarted at a dose of 0.5 mg/kg Q24h but discontinued due to adverse effects. A second 1.5 mg/kg epidural Depo-Medrone injection was administered, and low-dose PO prednisolone (0.1 mg/kg Q48h) was resumed with good long-term control. In dog 10, relapse occurred within one week of tapering prednisolone from Q12h to Q24h, with remission resumed after returning to the previous dose (0.8 mg/kg Q12h). Dog 13 remained on long-term prednisolone (approx 0.1 mg/kg Q24h), with recurrence of mild clinical signs upon dose reduction.

Follow-up information was available for all 15 dogs, with a mean follow-up duration of 25 months (range 12-50 months). A follow-up MRI was performed in 2 dogs. In dog 6, imaging at 4 months after diagnosis demonstrated a reduction in size of the previously thickened T1 nerve and nerve roots, with dimensions comparable to the contralateral, unaffected side. In dog 3, MRI at 25 months showed a reduction in size of the previously enlarged L7 nerve and roots.

## Discussion

This case series describes a group of dogs presenting with a variety of clinical signs of neurological disease, most commonly lameness and monoparesis, associated with diffuse, unilateral, or bilateral enlargement of nerves and nerve roots identified on advanced imaging. Numerous peripheral neuropathies have been reported in both veterinary and human medicine, many of which exhibit overlapping imaging features.^3^ A key finding in the present cases was diffuse enlargement involving the entire affected nerve and nerve roots, rather than focal or segmental changes and without associated spinal cord compression, as previously described.^4,7^ This uniform distribution is more consistent with an inflammatory process, whether infectious or non-infectious, than with a space-occupying lesion. However, infiltrative neoplastic diseases such as peripheral nervous system lymphoma, as well as metabolic, reactive, or inherited conditions, cannot be excluded.^3^

For the dog which underwent CT imaging at an external facility before presentation, the referring veterinarian reported thickening of the right L7 nerve roots with foraminal expansion. However, the images and full report were not available for review, so further details are limited. In contrast, in the 4 dogs that underwent a CT scan at the referral center, full-vertebral column CT revealed no relevant abnormalities; the only finding was incidental DISH in 1 dog. This helped to distinguish these cases from degenerative,^6^ compressive,^6^ or disc-associated lesions.^7^

The demographic profile, comprising young to middle-aged dogs of various breeds, and the subacute to chronic and progressive course with occasional relapses, further supports an immune-mediated etiology. These features are broadly consistent with the human condition of chronic inflammatory demyelinating polyneuropathy (CIDP), particularly when considering the MRI findings of nerve and nerve root enlargement and contrast enhancement, which are characteristic imaging hallmarks of CIDP.^8^

Chronic inflammatory demyelinating polyneuropathy is the most common autoimmune disease, comprising a heterogeneous group of polyneuropathies in humans^9^ that can present in symmetric or asymmetric, focal or multifocal forms.^8^ Histopathologically, CIDP is characterized by onion bulb formations due to Schwann cell proliferation associated with a localized chronic inflammatory reaction.^10^ Similar lesions in dogs are rare and more focal in distribution, typically involving a single nerve and roots and marked by chronic lymphocytic inflammation, in which onion bulb formation is not a feature.^3^ Although CIDP is not proposed as a definitive diagnosis in the dogs included in this study, the clinical and imaging parallels are noteworthy and provide a useful comparative framework for interpretation. These findings suggest that immune-mediated peripheral neuropathies in dogs may be more heterogeneous and potentially more common than previously recognized. The main clinical features, in terms of similarities and differences, between human CIDP and our dogs with PSRRN are summarized in Tables 1 and 2. Our dogs exhibited signs consistent with a focal, asymmetric variant, including lameness, monoparesis, reduced spinal reflexes, and postural reaction deficits, often accompanied by limb pain on manipulation. Muscle atrophy and sensory deficits were common, and 1 dog demonstrated cranial nerve involvement. These findings highlight the phenotypic variability observed and suggest that canine immune-mediated radiculoneuropathies, similar to CIDP in humans, might present in multiple clinical forms.

**Table 1:** Comparison of CIDP with dog radiculonerutis. Similarities.

Feature	Human CIDP	Dog radiculoneuritis (our data)
**Nerve root involvement**	MRI shows enlarged, contrast-enhancing nerve roots^2^	MRI shows enlarged, contrast-enhancing nerve roots
**Motor/Sensory deficits**	Weakness, paraesthesia, sensory loss^2^	Weakness, ataxia, lameness, pain
**Muscle atrophy**	Common^12^	Common
**Reflex abnormalities**	Reduced or absent^2^	Reduced or mildly increased
**Steroid responsiveness**	Corticosteroids are effective in many patients^2^	Several cases responded to oral prednisolone or epidural depomedrone
**CSF analysis**	CSF often shows elevated protein levels^2^	CSF often shows elevated protein levels

Abbreviations: CIDP = chronic inflammatory demyelinating polyneuropathy; CSF = cerebrospinal fluid; MRI = magnetic resonance imaging.

**Table 2:** Comparison of CIDP with dog radiculoneurtis. Differences.

Feature	Human CIDP	Dog radiculoneuritis (our data)
**Time course**	Symptoms develop over ≥ 2 months^2^	Onset ranged from 4 days to 6 months
**Symmetry**	Typically symmetric, both proximal and distal muscles weakness^2^	Often asymmetric or unilateral
**Age of onset**	Most common in adults (30-60 years)^2^	Predominantly young to middle-aged dogs

Abbreviation: CIDP = chronic inflammatory demyelinating polyneuropathy.

Infectious causes were considered unlikely based on negative serological and CSF testing, although could not be definitively excluded. CSF analysis, performed in 5 dogs, revealed albuminocytological dissociation in 4, a common finding in inflammatory neuropathies, including CIDP.^8^

Electromyography, performed in one dog, did not reveal abnormalities. However, the limited use of EMG in this cohort and the known variability of the technique in detecting proximal or patchy lesions reduce the diagnostic value of this single result. Nevertheless, EMG represent a useful adjunctive diagnostic tool in future cases, especially when combined with other electrophysiological or imaging modalities.

Standard treatment for CIDP in humans includes administration of corticosteroids, administration of IV immunoglobulin (IVIg), and plasmapheresis. IVIg is often used for long-term control. Newer immunotherapies, such as rituximab, target B cells, particularly in patients with autoantibodies.^8^ Our dogs were initially treated with meloxicam, resulting in little to partial improvement, except for one that responded well and another that relapsed shortly after discontinuation. All dogs, except one, were administered corticosteroids at doses ranging from anti-inflammatory to immunosuppressive. Clinical improvement was observed in all treated dogs, with complete clinical remission achieved by the end of the treatment period in all but 1 dog, which exhibited persistent Horner syndrome. The variability in treatment response supports the concept of a spectrum of disease severity, ranging from dogs requiring high-dose immunosuppressive prednisolone to others that improved with NSAIDs alone. Such variability mirrors what is observed in SRMA, where, although it’s termed “steroid-responsive,” mildly affected dogs can sometimes resolve with anti-inflammatory treatment alone.^11^

Repeat MRI was available in 2 cases. In 1 dog, 4 months, and in the other, 25 months after the initial diagnosis. In both dogs follow-up imaging demonstrated that the size of the nerve and nerve roots was reduced to be comparable with the contralateral, unaffected side, which proved particularly useful for assessing response to therapy and differentiating these cases from neoplastic causes. While the repeat imaging findings indicate a reduction in the caliber of the affected nerve and nerve roots, interpreting the initial thickening as an inflammatory process is based on the combination of clinical signs, therapeutic response, and imaging changes. In cases where follow-up imaging was not available, similar conclusions are drawn cautiously from the clinical course and therapeutic response.

Two dogs remained on a low maintenance dose of prednisolone, and 1 exhibited persistent Horner syndrome, findings suggestive of residual demyelination or chronic nerve injury, as can be seen in immune-mediated neuropathies.

Histopathology represents the definitive diagnostic modality for differentiating inflammatory from neoplastic conditions. However, obtaining histopathological confirmation would have required invasive surgical procedures associated with substantial morbidity. In this study, all dogs demonstrated a favorable and sustained response to medical management. As a result, pursuing invasive diagnostic procedures was not clinically justified. As all dogs showed improvements following anti-inflammatory medication, a presumptive diagnosis of inflammatory radiculoneuritis is considered most likely.

In a case report of unilateral, confirmed chronic hypertrophic ganglioneuritis in a dog,^3^ no response was observed with anti-inflammatory doses of corticosteroids, and surgical removal of the inflamed nerve root resulted in resolution of neurological deficits. In another study,^4^ symmetrical enlargements of the C2 nerve roots responded to immunosuppressive doses of corticosteroids, which places the emphasis on the favorable outcome of medical treatment rather than on surgical intervention or biopsy, which is rarely indicated. These findings are consistent with our study results.

In humans, CIDP has been linked with several comorbidities, including hepatitis C, lymphoma, HIV, transplants, connective tissue disorders, melanoma, and possibly diabetes mellitus.^8^ In our study, the caregivers were unaware of any traumatic incidents involving the dogs and no systemic comorbidities were identified at the time of diagnosis. Although 2 dogs presented with clinical signs of gastrointestinal disease before presentation, no association was found between the presenting complaint and clinical signs of gastrointestinal disease.

The limitations of this study include its retrospective design, small sample size, and lack of a control group. Only descriptive analyses were performed, limiting the ability to assess correlations or causality between treatment and outcomes. Relapse classification was based solely on clinical signs and treatment response, without repeat diagnostic testing, increasing the chance that some cases were misclassified and might have involved other central nervous system disorders. The main limitation is the lack of histopathological confirmation, which increases the risk of misdiagnosis in the cases included in this study and prevents us from reaching a definitive diagnosis of radiculoneuritis.

## Conclusion

In this study, we describe 15 medium- to large-breed dogs, predominantly young to middle-aged, with diffuse enlargement of spinal nerves and nerve roots involving the full extent of the affected roots. While differentiation from neoplastic disease was not possible based on clinical presentation and imaging findings alone, the favorable response to anti-inflammatory treatment and the clinical course observed during long-term follow-up supported a presumptive immune-mediated etiology in these cases. These findings highlight the importance of including PSRRN as a differential diagnosis in dogs presenting with nerve and nerve roots enlargement, as appropriate medical management can be associated with a favorable long-term clinical outcome.

## Off-label antimicrobial declaration

The authors declare no off-label use of antimicrobials.

## Institutional animal care and use committee or other approval declaration

This retrospective study involved client-owned dogs and was performed with informed owner consent.

## Abbreviations

CIDP: Chronic inflammatory demyelinating polyneuropathy
CT: computed tomography
DISH: diffuse idiopathic skeletal hyperostosis
NSAID: nonsteroidal anti-inflammatory drug
